# Odontogenic tumors in Nigerian children and adolescents- a retrospective study of 92 cases

**DOI:** 10.1186/1477-7819-2-39

**Published:** 2004-11-27

**Authors:** Oluseyi F Ajayi, Akinola L Ladeinde, Wasiu L Adeyemo, Mobolanle O Ogunlewe

**Affiliations:** 1 Department of Oral Pathology and Biology, College of Medicine, University of Lagos P.M. B. 12003, Lagos, Nigeria; 2 Department of Oral and Maxillofacial Surgery, College of Medicine, University of Lagos, P.M. B. 12003, Lagos, Nigeria

## Abstract

**Background:**

Tumours arising from odontogenic tissues are rare and constitute a heterogenous group of interesting lesions. The aim of this study was to determine the relative frequency of odontogenic tumors (OT) among Nigerian children and adolescents 19 years or younger.

**Patients and methods:**

The histopathology records were retrospectively reviewed for all the tumors and tumor-like lesions of the oral cavity and the jaws seen in children and adolescents ≤ 19 years seen between January 1980 and December 2003. Hematoxylin and eosin-stained sections were re-evaluated and the diagnosis in each case was confirmed or modified according to World Health Organization (WHO) classification, 1992; and were subjected to analysis of age, sex, site of tumor and histopathologic type.

**Results:**

A total of 477 tumors and tumor-like lesions were seen in patients ≤ 19 years during the period of the study. Of these, 92 (19.3%) were odontogenic tumors. Benign odontogenic tumors constituted 98.9% of the cases seen, while only 1 case (1.1%) of malignant variety was seen during the period. The mean (SD) age of patients was 14.9 (± 3.1) years (range, 4–19 years). Male-to-female ratio was 1:1; and mandible-to-maxilla ratio was 2.7:1. OT's were most frequently seen in patients aged 16–19 years (46.7%) and the least number (2.2%) were found in patients aged 0–5 years. Among nine histologic types of OT seen, ameloblastoma (48.9%), adenomatoid odontogenic tumor (19.6%) and odontogenic myxoma (8.7%) were predominant. Multicystic/solid and unicystic variants of ameloblastoma were diagnosed in 40 (89%) and 5 (11%) cases respectively.

**Conclusions:**

Odontogenic tumors are relatively common in children and adolescents in Nigeria. One out of every 5 children and adolescents with tumors and tumor-like lesions of oral cavity and the jaws seen in this study had a diagnosis of odontogenic tumor.

## Background

Tumors and tumor-like growths arising from the odontogenic tissues constitute a heterogenous group of particularly interesting lesions, as they display the various inductive interactions that normally occur among the embryologic components of the developing tooth germ [1]. In humans, tumors of odontogenic tissues are comparatively rare, comprising of about 1% of all jaw tumors [2]. In children and adolescents, neoplastic lesions are often benign and are of mesenchymal origin [3,4]. Choung and Kaban [5] were of the opinion that tumor histology in this age group did not correspond to their clinical behaviour.

There are few reports especially from Africa, which have specifically reported the high frequency of odontogenic tumors (OT) in children and adolescents in the literature. In most previous African reports, odontogenic tumours in children were presented as part of orofacial [3] or oral tumors in this age group [3,6]; or presented as specific tumours e.g. ameloblastoma or adenomatoid odontogenic tumor [7,8]. We could find only a single report on odontogenic tumors in children and adolescent in African environment [9]. The aim of this study was to determine the relative frequency of odontogenic tumors among children and adolescent ≤ 19 years seen over a period of 24 years (1980–2003).

## Patients and methods

The histopathology records of the Department of Oral Pathology and Biology, College of Medicine, University of Lagos, Lagos, Nigeria, were reviewed for all the tumors and tumor-like lesions of the oral cavity and the jaws seen in children and adolescent ≤ 19 years from January 1980 to December 2003. Hematoxylin and eosin-stained sections was re-evaluated and the diagnosis in each case was confirmed or modified according to World Health Organization (WHO) 1992 classification [10]. The data was subjected to analysis of age, sex, site of tumor and histopathologic type. The age of the patients was divided in four groups: Group 1 (0–5 years); group 2 (6–10 years); group 3 (11–15 years); group 4 (16–19 years). Data was analyzed using SPSS for Window (version 11.0; SPSS Inc., Chicago, IL) and frequency tables and cross tables were prepared.

## Results

A total of 477 tumors and tumor-like lesions were seen in patients ≤ 19 years during the period of the study. Of these, 92 (19.3%) were odontogenic tumors. Benign odontogenic tumors constituted 98.9% of the cases seen, while only 1 case (1.1%) of malignant variety was seen during the period. Table 1 shows the various histologic types and their relative frequency. There were 47 males and 45 females; a male-to-female ratio of 1:1 was observed. The mean (SD) age of patients was 14.9 (± 3.1) years (range, 4–19 years). Most of the patients (46.7%) were in age group 4 and the least number of patients were found in age group 1 (2.2%) Table 2.

**Table 1:** Relative frequency of odontogenic tumors in children and adolescents (≤ 19 years)

**Histological Types**	**Frequency (%)**
Ameloblastoma	45 (48.9)
Calcifying epithelial odentogenic tumor	1(1.1)
Ameloblastic fibroma	5 (5.4)
Adenomatoid odontogenic tumor (AOT)	18 (19.6%)
Calcifying odontogenic cyst (COC)	3 (3.3)
Odontoma	4 (4.3)
Odontogenic fibroma	7 (7.6)
Myxoma	8 (8.7)
Ameloblastic carcinoma	1 (1.1)
Total	92 (100)

**Table 2:** Age distribution of patients with odontogenic tumors (years)

**Histological Types**	**Group 1**	**Group 2**	**Group 3**	**Group 4**	**Total**
	**Age 0–5**	**Age 6–10**	**Age 11–15**	**Age 15–19**	
Ameloblastoma	1	2	22	20	45
Calcifying epithelial odontogenic tumour	0	0	1	0	1
Ameloblastic fibroma	0	1	1	3	5
Adenomatoid odontogenic tumor (AOT)	0	3	6	9	18
Calcifying odontogenic cyst	0	2	1	0	3
Odontoma	0	1	1	2	4
Odontogenic fibroma	1	0	2	4	7
Myxoma	0	1	4	3	8
Ameloblastic carcinoma	0	0	0	1	1
Total	2	10	38	42	92

Table 3 shows the gender and site distribution of various histologic typing of odontogenic tumors. The tumors occurred more often in the mandible (67) than in the maxilla (25) giving a maxilla-to-mandible ratio of 1:2.7.

**Table 3:** Distribution of histologic types of odontogenic tumors according to gender and site of tumor

**Histological type**	**Number (%)**	**Gender**		**Site**	
		**Male**	**Female**	**Mandible**	**Maxilla**

Ameloblastoma	45 (48.9)	28	17	40	5
Calcifying epithelial odontogenic tumor	1 (1.1)	1	0	0	1
Ameloblastic fibroma	5 (5.4)	1	4	5	0
Adenomatoid odontogenic tumor	18 (19.6)	6	12	8	10
Calcifying odontogenic cyst	3 (3.3)	2	1	1	2
Odontoma	4 (4.3)	2	2	0	4
Odontogenic fibroma	7 (7.6)	5	2	5	2
Myxoma	8 (8.7)	2	6	7	1
Ameloblastic carcinoma	1 (1.1)	0	1	1	0

*Ameloblastoma *constituted almost half (48.9%) of the odontogenic tumors with female-to-male and maxilla-to-mandible ratios of 1:1.7 and 1:8 respectively. The mean (SD) age of patients in this group was 15.1 (± 3.0) years (range, 4–19 years) with most patients (49%) in age group 3. Multicystic/solid and unicystic variants were diagnosed in 40 (89%) and 5 (11%) cases respectively.

*Adenomatoid odontogenic tumor *(AOT) was the second most common tumor in this series (Table 1) accounting for 19.6% of odontogenic tumors in this population. All the patients were between 8 and 19 years (Mean ± SD; 14.6 ± 3.2) with most of them (50%) in age group 4. More females (12) were affected than males (6); a male-to-female ratio of 1:2 was observed. Maxillary lesions (10) were commoner than mandibular lesions (8).

*Odontogenic myxoma *accounted for 8 cases (8.7%) of odontogenic tumors with a male-to-female ratio of 1:3. Only 1 case occurred in the maxilla, the rest (7) were found in the mandible. The patients were found between 10 and 19 years.

*Odontogenic fibroma *accounted for 7 cases (7.6%) of tumors in this series. More cases were found in males and in the mandible. Patients were seen between 5 and 19 years in this group.

*Ameloblastic fibroma *accounted for 5.4% of cases seen. The lesion occurred exclusively in the mandible with a male-to-female ratio of 1:4.

*Odontomas *accounted for 4.3% of OT seen. They were exclusively found in the maxilla with equal sex predilection.

Table 3 shows the gender and site distribution of other less common odontogenic tumors in children and adolescent ≤ 19 years of age.

## Discussion

We present a report of odontogenic tumours in children and adolescents aged ≤ 19 years. This report represents the largest series of odontogenic tumors in children and adolescents in Africa. We found that 19.3% of tumors and tumor-like lesions of the oral cavity and the jaws in children and adolescent in this study were odontogenic tumors. This is similar to the findings of Arotiba [6]. However, other authors have reported higher [9,11-13] or lower [4,14] frequency of these heterogenous group of lesions in children and adolescents.

A major problem in comparing our report with previous studies is the lack of uniformty in the age group studied in those reports. Some studies were restricted to children under the age of 14 years [3,15], or 15 years [4,6,11-13,16], while others included higher age groups [9,14]. Odontogenic tumors were most frequently seen in patients in group 4 in this study (> 15 years) in agreement with Adebayo *et al *[9]. Al-Khateeb *et al *[14] reported that odontogenic tumors were most commonly (72%) seen in patients aged 12–18 years in their report. Other authors have reported that odontogenic tumors were most frequently seen in patients aged 11–15 years [4,11,13]; these authors, however considered patients aged 0–15 years in their studies.

Odontogenic tumors were less frequently seen in patients aged 0–5 years in this study in agreement with most reports in the literature [4,9,11-14,17]. About 98% of OT in the present series was found in patients older than 5 years. Many odontogenic tumors are thought to arise from the tooth germ [18]. In most permanent teeth, crown formation is completed by the age of 4 or 5 years; odontogenic tumors seemed to develop after crown formation [12,13]. This strengthens the impression that the majority of odontogenic tumors arise from quiescent remnants of the tooth germ [14].

Odontogenic tumors in children are known to have predilection for the mandible [11-13]. This is also corroborated by our findings with 73% of the tumors in this series found in the mandible. Al-Khateeb *et al *[14] however found 64% of OT in the maxilla. Odontomas were exclusively found in the maxilla in the present series. Males were slightly affected by OT in our study in agreement with Adebayo *et al *[11]; whereas Ulmansky *et al *[4] reported female preponderance in their study.

Ameloblastoma was the most common tumor in this study in agreement with reports from Africa [6,11]. Other authors reported odontoma as the most frequently seen OT in children [12-14]. Ulmansky *et al *[4] found odontogenic myxoma as the most common OT in children in their study. The gender and site distribution of ameloblastoma in this study is in agreement with other reports [6,11-14]. Ameloblastoma was found in all the age groups considered in this study, unlike other histologic types of OT. Few cases of unicystic variant of ameloblastoma (11%) were seen in the present series. Unicystic ameloblastoma has been reported to be more common in Western children than African children [19]. Previous data from Africa as corroborated by the present series, have shown a low percentage of unicystic ameloblastomas in their patient population compared to other parts of the World [20,21]. Unicystic tumors have a different prognosis to the multi cystic type and are said to be more common in children [19,22,23].

Adenomatoid odontogenic tumor (AOT) was the second most common OT in this study and 50% of this tumor was found in patients >15 years. Asamoa [3] reported AOT as the most frequent pediatric odontogenic tumor in Nigeria; whereas Arotiba [6] found AOT as the second most common OT after ameloblastoma in Nigerian children. The relative frequency of 19.6% found in this study is higher than in other reports [6,9,13,14]. More of this lesion was found in the maxilla in concordance with previous reports [6,7,9,24,25]. OT is reported to be more common in females [1,7,17,26]; and this is also confirmed by our findings.

In children, the reported incidence of myxoma ranges from 1.2% to 39% [4,6,9,13]. Myxoma was the third most common OT in this study with an incidence of 8.7%. Ulmansky *et al *[4] reported that odontogenic myxoma was the most common OT in Israeli children. The gender predilection favors females in previous publications [4,9,24,26,27]. This study found that the male-to-female ratio was 1:3, in agreement with previous reports. Previous publications [9,24,27] reported that both jaws were equally affected in their reports; a ratio of 1:7 was found in the maxilla and mandible respectively in the present study. Odukoya [26] also found maxilla-to-mandible ratio of 1:3.

Odontogenic fibroma was reported to be rare in children with incidence ranging from 0% to 1.3% of OT [4,9,11,13]. An incidence of 7.8% found in our study was however, similar to that of Al-Khateeb *et al *[14]. Arotiba [6] reported that 12.5% of odontogenic tumors in his study were odontogenic fibroma. More cases were found in males and in the mandible in agreement with the report of Lu *et al *[17]; whereas others [1,26] have reported females and maxillary predilection. Ameloblastic fibroma was exclusively seen in the mandible, accounting for 5.4% of OT seen in this study. Females were also affected more than males in the ratio of 1:4. Other authors [17,26] have also reported predilection of ameloblastic fibroma for the mandible but with both jaws affected equally.

Odontomas are often regarded as dental hamartomas, rather than odontogenic neoplasms [14,28,29]. Odontoma is relatively rare in Nigerian children as confirmed in our report and others from Nigeria [6,9,11], accounting for 4.3% of OT in this study. This contrasts the findings of Al-Khateeb *et al *[14], Tanaka *et al *[12] and Sato *et al *[13] that reported odontoma as the most frequently seen OT in North Jordanian and Japanese children respectively. Most odontomas are discovered on routine radiograph and do not produce clinical symptoms [1]. This may be responsible for the low incidence observed in African population, because most patients in our environment do not seek medical consultation unless there are symptoms suggesting an obvious pathology. While some authors reported that odontomas commonly affect the mandible [12,17], others have reported predilection for the maxilla [1,13,14] and some authors have reported equal distribution in both jaws [9,11,25]. Odontomas were found exclusively in the maxilla in the present series.

Malignant odontogenic tumors are rare, most especially in children [1,4,6,9,11,12,15,30]. No cases of malignant odontogenic tumors were found in African children and adolescents [6,9,11,30], Israeli children [4], North Jordanian children and adolescents [14] and Japanese children [12,13]. A case ameloblastic carcinoma of the mandible in a 16-year old girl was, however seen in this study.

## Conclusions

Odontogenic tumors are relatively common in children and adolescents in Nigeria. One out of every 5 children and adolescents with tumor and tumor-like lesions of oral cavity and the jaws seen in this study had a diagnosis of odontogenic tumor.

## Competing interests

The author(s) declare that they have no competing interests.

## Authors' contribution

**OFA **conceived the study, coordinated the write-up and submission of the article, and also reviewed the slides.

**ALL**, **WLA **and **MOO **did the literature search and participated in the writing of the manuscript.

All the authors read and approved the final manuscript.

## Funding Source

None declared
